# Challenges in understanding the impact of blood pressure management on cerebral oxygenation in the preterm brain

**DOI:** 10.3389/fphys.2012.00471

**Published:** 2012-12-18

**Authors:** Aminath Azhan, Flora Y. Wong

**Affiliations:** ^1^The Ritchie Centre, Monash UniversityMelbourne, VIC, Australia; ^2^Monash Newborn, Monash Medical CentreMelbourne, VIC, Australia; ^3^Department of Pediatrics, Monash UniversityMelbourne, VIC, Australia

**Keywords:** infants, newborn, preterm, hypotension, cerebral oxygenation

## Abstract

Systemic hypotension in preterm infants has been related to increased mortality, cerebrovascular lesions, and neurodevelopmental morbidity. Treatment of hypotension with inotropic medications aims at preservation of end organ perfusion and oxygen delivery, especially the brain. The common inotropic medications in preterm infants include dopamine, dobutamine, adrenaline, with adjunctive use of corticosteroids in cases of refractory hypotension. Whether maintenance of mean arterial blood pressure (MAP) by use of inotropic medication is neuroprotective or not remains unclear. This review explores the different inotropic agents and their effects on perfusion and oxygenation in the preterm brain, in clinical studies as well as in animal models. Dopamine and adrenalin, because of their α-adrenergic vasoconstrictor actions, have raised concerns of reduction in cerebral blood flow (CBF). Several studies in hypotensive preterm infants have shown that dopamine elevates CBF together with increased MAP, in keeping with limited cerebro-autoregulation. Adrenaline is also effective in raising cerebral perfusion together with MAP in preterm infants. Experimental studies in immature animals show no cerebro-vasoconstrictive effects of dopamine or adrenaline, but demonstrate the consistent findings of increased cerebral perfusion and oxygenation with the use of dopamine, dobutamine, and adrenaline, alongside with raised MAP. Both clinical and animal studies report the transitory effects of adrenaline in increasing plasma lactate, and blood glucose, which might render its use as a 2nd line therapy. To investigate the cerebral effects of inotropic agents in long-term outcome in hypotensive preterm infants, carefully designed prospective research possibly including preterm infants with permissive hypotension is required. Preterm animal models would be useful in investigating the relationship between the physiological effects of inotropes and histopathology outcomes in the developing brain.

## Introduction

Systemic hypotension is a relatively common complication of preterm birth, affecting approximately one-third of very low birth weight infants. Systemic hypotension has been associated with increased mortality, intraventricular hemorrhage, periventricular leukomalacia, and neurodevelopmental morbidity in the preterm infant (Miall-Allen et al., 1987; Watkins et al., 1989; Goldstein et al., 1995; Cunningham et al., 1999), although a causal relationship has yet to be established. Given the risks of hypotension, preterm infants identified as “hypotensive” often receive treatments to elevate the blood pressure, with 16–52% receiving volume expansion (Al-Aweel et al., 2001) and up to 53% (Laughon et al., 2007) receiving inotropic agents for hypotension, with the most common drugs of choice being dopamine, dobutamine, and adrenaline.

Several studies have shown that systemic hypotension is associated with reduced end-organ perfusion, low cerebral blood flow (CBF), and cerebral oxygen delivery, which are presumably the mechanism for the adverse neurodevelopmental outcome associated with hypotension (Lou et al., 1977; Tsuji et al., 2000; Munro et al., 2004). Therefore, the main goal for maintaining mean arterial blood pressure (MAP) in preterm infants is to preserve organ perfusion and oxygen delivery, particularly the brain. However, the inotropic medications selected to raise MAP are obviously those which are known to act primarily on the cardiovascular system, their effects on the cerebral vasculature and oxygen delivery are less known. In this review, we have described and synthesized the research literature, in both animal and clinical studies, on the impact of inotropic agents on cerebral perfusion and oxygenation in the preterm brain. We have also identified the knowledge gap in the literature, and the future research required to address the issues and inform clinical practice.

## Effects/implication of hypotension in preterm infants

Although there are epidemiological data for “normative” blood pressure values in preterm infants, the ranges of normal blood pressure where adequate organ perfusion is ensured for different gestational and postnatal ages remain unclear. Several studies have attempted to create reference ranges for blood pressure in neonates based on birthweight, gestational age, and/or postnatal age (Watkins et al., 1989; Cunningham et al., 1999; Lee et al., 1999). Data from these studies provided support for the commonly cited “rule of thumb” that defines hypotension as MAP below an infant's gestational age in weeks, using the lower 90% confidence interval of MAP as the threshold for hypotension (BAPM, 1998; Cunningham et al., 1999). Hypotensive infants will often receive treatment for restoration of MAP to the target reference range, as many studies have found that systemic hypotension is associated with increased brain injury and poor neurodevelopmental outcome (Goldstein et al., 1995; Seri and Evans, 2001). The brain of the preterm infants is particularly vulnerable to decreases in perfusion pressure, as several reports (Munro et al., 2004; Wong et al., 2008) have found impaired cerebral autoregulation in preterm infants. The direct variation of CBF and MAP in preterm infants implies hypotension would lead to low CBF and the risk of hypoxic-ischemic brain injury. The risk is accentuated in the preterm white matter, possibly related to the development of the vascular supply to these regions. The cerebral white matter is supplied by long and short penetrating arteries, and the distal fields of these vessels are not yet fully developed in the preterm brain, thus forming vascular end zones where perfusion is thought to be relatively close to the metabolic demand (Takashima and Tanaka, 1978; Volpe, 2001). The preterm cerebral white matter at these vascular end zones are most sensitive to falls in cerebral perfusion pressure (Borch et al., 2010), and it is therefore widely thought that these zones would suffer from severe ischemia with decreases in CBF. In addition, CBF is markedly lower in the preterm white matter compared to the cortical gray matter (Borch and Greisen, 1998), further increasing the vulnerability of the white matter to hypoxic-ischemic injury. Also, an association between hypotension and cerebrovascular lesions has been described (Miall-Allen et al., 1987; Watkins et al., 1989; Bada et al., 1990; Cunningham et al., 1999), supporting the notion that cerebral ischemic injury occurs secondary to failure of autoregulation to maintain CBF during hypotension. The implication is that pharmacological manipulation of systemic blood pressure is likely to affect cerebral perfusion pressure and CBF.

While systemic hypotension in preterm infants is often rigorously treated with the goal of restoring CBF and minimizing cerebral injury, the association between treatment of hypotension and improved outcome is still controversial (Cunningham et al., 1999; Limperopoulos et al., 2007). Whether maintenance of MAP by use of inotropic medication is neuroprotective, or indeed might lead to cerebral compromise, remains unclear. There is no evidence that treatment of systemic hypotension results in short- or long-term improvement of neurological outcomes (Dempsey and Barrington, 2007), and such evidence could be obtained only carefully designed prospective studies. It is also not known whether the use of dopamine, dobutamine, or adrenaline as the first-line vasoactive/inotropic agent, in the treatment of the hypotensive preterm infant, offers an advantage over each other in improving outcome.

## Blood pressure management in preterm infants

There is considerable variation between neonatal centers in the reported prevalence of hypotension, the threshold for therapeutic intervention, and the choice of cardiovascular support (Al-Aweel et al., 2001). In a cohort of 1507 infants born between 23 and 27 weeks of gestational age at 14 centers (Laughon et al., 2007), the proportion of infants who received inotropic medication for hypotension varied between 6 and 64% in the different centers, and uniform thresholds of blood pressure for commencing inotropic treatment were not apparent. The inter-center variations could not be explained by variations in infant characteristics, and are more likely to reflect differences in care practices (Laughon et al., 2007).

The most commonly employed therapies are volume expansion, followed by use of inotropic agents if hypotension persists. Volume expansion with colloid or crystalloid is frequently used as a first-line treatment in neonatal hypotension. Even in the absence of hypovolemia, volume expansion has the potential to increase cardiac output and blood pressure through the Frank–Starling mechanism and may therefore be a useful therapeutic strategy to avoid use of inotropic medications (Subhedar, 2003). It is less effective than inotropic medications at increasing blood pressure (Lundstrom et al., 2000). In cases of refractory or vasopressor-resistant hypotension, corticosteroid is often added as an adjunctive therapy (see below for further discussion).

Dopamine is the most commonly used inotropic medication in the treatment of neonatal hypotension. It is an endogenous catecholamine precursor of noradrenaline with sympathomimetic properties. It exerts cardiovascular effects through direct stimulation of dopaminergic, adrenergic and serotonic receptors, or indirectly by stimulating adrenergic receptors though its conversion to noradrenaline in sympathetic nerve endings (Subhedar, 2003). The cardiovascular actions of dopamine depend on the overall balance of dopaminergic, α- and β-receptors agonist activity, and are likely to be dose-related. The increase in systemic blood pressure following dopamine administration is thought to be a result of arterial vasoconstriction and increased cardiac output (Seri and Evans, 2001). There is wide inter-individual variation in the blood pressure response to dopamine infusion, but whether this reflects differences in plasma concentration, underlying pathophysiology, or peripheral vs. cardiac actions remain unknown. Most hypotensive infants respond to doses of 2.5–20 μg.kg^−1^.min^−1^, with vast majority of very low-birthweight infants responding to doses <10 μg.kg^−1^.min^−1^(Gill and Weindling, 1993; Roze et al., 1993; Lundstrom et al., 2000). Treatment of neonates with high-dose dopamine (>20 μg.kg^−1^.min^−1^) is usually avoided because of concerns regarding excessive α-receptor-mediated peripheral vasoconstriction, with a corresponding reduction in cardiac output (Roze et al., 1993). Addition of another agent such as dobutamine is preferred by many clinicians. On the other hand, there is no evidence that, when required to normalize blood pressure, high-dose dopamine treatment has detrimental vasoconstrictive effects (Seri and Noori, 2005).

Dobutamine is a synthetic analogue of isoprenaline with some chemical similarities to dopamine. Dobutamine stimulates both α- and β-adrenergic receptors but is relatively selective for cardiac β-1 receptors for its inotropic effects, with lower affinity for peripheral α-1 and β-2 receptors (Crocker, 1994). In neonates, positive effects on left ventricular performance at dobutamine doses of 5–10 μg.kg^−1^.min^−1^(Stopfkuchen et al., 1987) and increases systemic blood flow, as measured by flow in the superior vena cava (SVC), at doses of 10–20 μg.kg^−1^.min^−1^ have been demonstrated (Osborn et al., 2002). Randomized trials comparing the effectiveness of dopamine versus dobutamine in increasing blood pressure showed that dobutamine was less effective than dopamine at increasing blood pressure in hypotensive preterm infants (Subhedar and Shaw, 2003). However, dobutamine has a superior effect in maintaining left ventricular output in these infants (Roze et al., 1993; Subhedar and Shaw, 2003). No significant difference was found between the effects of dopamine and dobutamine for infant mortality, or the incidence of cerebrovascular lesions. However, the impact of dopamine compared to dobutamine, when used as inotropic agents for blood pressure support, on long-term neurodevelopmental outcome is still uncertain (Subhedar and Shaw, 2003).

Systemic blood flow, as Doppler measurement of SVC flow, has been proposed to be an index of brain perfusion and a low SVC flow of <41 ml/kg/min as the threshold for commencing inotropic medications (Kluckow and Evans, 2000a,b; Osborn et al., 2002). When SVC flow was used as the treatment criteria and the outcome measure, dobutamine was found to be more effective than dopamine in raising SVC flow (Osborn et al., 2002). However, the relationship between SVC flow and CBF is complex because SVC flow includes venous return from many extracerebral vascular beds and in which the regulation of blood flow may differ significantly from that of brain, especially in pathophysiological conditions. For example, with hypovolemia and hypotension, peripheral vasoconstriction occurs to maintain systemic blood pressure and cerebral perfusion pressure, while cerebral vasodilatation occurs to increase CBF (Malcus et al., 1991). The opposite changes in peripheral and CBF thus render interpretation of SVC flow difficult. Long-term effect of inotropic treatment in infants with low SVC flow is also unclear. In a randomized controlled trial in which infants with low SVC flow were treated with dopamine or dobutamine, there was a trend toward increased death in the dobutamine group; there was a trend toward increased neurodevelopmental disability in the dopamine group. However, the study was difficult to interpret because of the absence of a control group of infants with untreated low SVC flow for comparison (Osborn et al., 2007).

Milrinone is a selective inhibitor of type III cAMP phosphodiesterase isoenzyme in cardiac and vascular muscle. It has both positive inotrope and vasodilator effects (Chang et al., 1995). Milrinone has been used therapeutically (Chang et al., 1995; Ramamoorthy et al., 1998; Duggal et al., 2005) and prophylactically (Hoffman et al., 2003) to improve low cardiac output in infants after cardiac surgery. However, a main adverse effect of milrinone is actually hypotension, which usually precludes its use alone in the setting of neonatal hypotension. Milrinone was trialed as a prophylactic treatment to prevent low SVC flow in very preterm infants but was found not to be effective (Paradisis et al., 2009).

Adrenaline (epinephrine) is often used as a second or third line of treatment in systemic hypotension, usually in addition to dopamine and/or dobutamine infusion. Adrenaline acts upon α1-, β1- and β2-adrenergic receptors. In a randomized control trial in very low-birthweight infants with early postnatal hypotension, in whom either adrenaline or dopamine infusion was administered (Valverde et al., 2006), low-dose adrenaline is as effective as low/moderate-dose dopamine in increasing systemic MAP and has a more intense chronotropic effect. Importantly, the adrenaline-treated infants did not differ with respect to short- and medium-term outcomes including neurodevelopment, when compared with dopamine-treated infants (Pellicer et al., 2009). Furthermore, infants who gained a normalized blood pressure with either dopamine or adrenaline had neurodevelopmental outcomes comparable with those of control normotensive infants (Pellicer et al., 2009). However, adrenaline has the transitory effect of increasing plasma lactate, blood glucose and the need for insulin therapy (Valverde et al., 2006), probably due to β2-adrenoreceptor stimulation, which therefore raises concern about the use of adrenaline as a first-line inotrope in preterm infants prone to such metabolic disturbances.

Although the majority of preterm neonates will achieve blood pressure values perceived to be normal with volume expansion, vasopressor and/or inotrope administration, up to a quarter of all very low birth weight neonates treated for hypotension do not respond to moderate-to-high dose of vasopressor administration (Ng et al., 2004). The etiology of vasopressor-resistant hypotension is thought to be a combination of transient adrenocortical insufficiency of prematurity and downregulation of the cardiovascular adrenergic receptors (Seri, 2006a). In these patients, blood pressure may increase with corticosteroid administration. Raised corticosteroid levels increase intracellular calcium availability in the cardiovascular system, leading to increased myocardial and vascular smooth muscle cell contractility in response to catecholamines (Wehling, 1997). Corticosteroid would also act via enhancement of adrenergic, dopaminergic, and angiotensin II receptor expression, thus sensitizing the cardiovascular system to endogenous and exogenous catecholamines. In preterm infants who develop vasopressor dependence or vasodepressor-resistant hypotension, low doses of hydrocortisone have been shown to result in improvement of blood pressure without compromising cardiac function or systemic perfusion and therefore allow vasopressor support to be reduced over the next 24–72 h (Helbock et al., 1993; Ng et al., 2001; Seri and Evans, 2001; Ng et al., 2004; Seri and Noori, 2005; Seri, 2006a,b). Unlike dexamethasone, hydrocortisone has not been shown to be associated with neurodevelopmental delay or cerebral palsy (Lodygensky et al., 2005; Karemaker et al., 2006; Rademaker et al., 2007). However, caution must be exercised as available data suggest that co-exposure of hydrocortisone and indomethacin in the first postnatal week, in very preterm infants being treated for patent ductus arteriosus, increases the risk of spontaneous intestinal perforation (Watterberg et al., 2004).

## Specific cerebral effects of inotropic agents in the immature brain (Table 1)

### Cerebral blood flow in preterm infants treated with inotropic agents

The effect of dopamine on maintaining CBF has been the subject of many clinical and experimental studies; however, no consensus has been reached regarding the efficacy or safety of dopamine administration for this purpose (Munro et al., 2004; Paradisis et al., 2009). Because of its α-adrenergic vasoconstrictor actions, there has been concern that dopamine might exert a direct vasoconstrictor effect on the immature cerebral vasculature, so impairing CBF (Munro et al., 2004). Such an effect would be compounded by the already inadequate perfusion and oxygen delivery to the brain that may be the result of the pre-existing hypotension. However, several studies in hypotensive preterm infants have shown that dopamine elevates CBF together with increased MAP, consistent with the limited autoregulation of CBF that is characteristic of infants at this age (Seri et al., 1993; Munro et al., 2004; Pellicer et al., 2005; Wong et al., 2008). The increase in CBF following dopamine administration was greater in hypotensive preterm infants compared to normotensive infants, possibly because the hypotensive infants might have more severe impairment of CBF autoregulation (Seri et al., 1993, 1998; Zhang et al., 1999; Lundstrom et al., 2000; Jayasinghe et al., 2003; Munro et al., 2004).

**Table 1:** **Summary of systemic and cerebral effects of inotropic agents**.

**Inotropic agent**	**Mechanism of action**	**Dose-dependant effects**	**Changes in CBF (clinical and animal studies)**	**Changes in cerebral metabolic rate (animal studies)**
Dopamine	Dose-related action on adrenergic α and β, and dopaminergic receptors	Low dose (0.5–4 μg.kg^−1^.min^−1^): renal vasodilation, direct renal tubular effect, positive inotropy	↑ CBF with ↑ MAP in hypotensive preterm infants (Seri et al., 1993; Jayasinghe et al., 2003; Munro et al., 2004; Pellicer et al., 2005)	Stimulation of cerebral dopaminergic receptors in adult baboons
		Medium dose (4–10 μg.kg^−1^.min^−1^): positive inotropy, vasocontriction	↑ CBF/cerebral oxygenation in both preterm and term newborn piglets (Ferrara et al., 1995; Nachar et al., 2011)	↑ cerebral oxygen consumption with ↑ CBF (McCulloch and Harper, 1977)
		High dose (>8–10 μg.kg^−1^.min^−1^): positive inotropy and chronotropy	Cerebrovasodilatation with ↑ CBF in adult animal studies (Von Essen, 1974; Myburgh et al., 2002)	
Dobutamine	Relatively selective for cardiac β-1 receptors, with lower affinity for peripheral α-1 and β-2 receptors	Low-medium dose (5–10 μg.kg^−1^.min^−1^): positive inotropy and chronotropy, peripheral dilatation	↑ Superior vena cava flow in preterm infants, no data on CBF (Osborn et al., 2002)	↑ Systemic oxygen consumption and offset systemic oxygen delivery (Penny et al., 2001)
		Medium-to-high dose (>10 μg.kg^−1^.min^−1^): positive inotropy and chronotropy, vasoconstriction.	↑ Cerebral oxygenation with ↑ MAP in term newborn piglets (Nachar et al., 2011)	
			↑CBF in term newborn piglets, but not preterm piglets (Ferrara et al., 1995)	
Adrenaline	Acts on adrenergic α-1, β-1 and β-2 receptors	Low-moderate dose (0.02–0.1 μg.kg^−1^.min^−1^): positive inotropy and chronotropy, peripheral (renal and mesenteric) vasodilatation, metabolic effects	↑ CBF with ↑ MAP in hypotensive preterm infants (Pellicer et al., 2005)	–
		Moderate-high dose (>0.1 μg.kg^−1^.min^−1^): vasoconstriction in addition to effects above	↑ Cerebral oxygenation with ↑ MAP in newborn piglets (Nachar et al., 2011)	
Noradrenaline	Mainly acts on adrenergic α-1, α-2 receptors	Peripheral vasoconstriction	↑ Cerebral oxygenation with ↑ MAP in newborn piglets (Nachar et al., 2011)	–
Milrinone	Type 3 cAMP phosphor-diesterase inhibitor	Positive inotropy and peripheral vasodilatation	No effect on cerebral oxygenation or MAP in newborn piglets (Nachar et al., 2011)	–

*CBF, cerebral blood flow; MAP, mean arterial pressure*.

A randomized clinical study comparing dopamine- and adrenaline-treated hypotensive preterm infants revealed that low-dose adrenaline or low/moderate-dose dopamine both raised cerebral perfusion, cerebral blood volume (CBV), and oxygenation (Pellicer et al., 2005). Notably, the CBV and cerebral intravascular oxygenation increased in parallel, suggestive of a vasodilatory effect on cerebral vasculature, and/or the recruitment of unperfused capillaries (Pellicer et al., 2005).

### Animal studies investigating cerebral blood flow with inotropic agents

In adult animal studies, dopamine infusion was shown to result in cerebral vasodilation with increased CBF (Von Essen, 1974). In anesthetized adult sheep (Myburgh et al., 2002), epinephrine, norepinephrine, and dopamine all increased CBF from baseline, together with increase in blood pressure, but this is likely to be due to anesthesia-related autoregulatory impairment. In contrast, in the awake adult sheep (Myburgh et al., 2002), cerebral autoregulation preserves a relatively constant CBF despite the increase in systemic blood pressure with epinephrine or norepinephrine infusion. However, of great importance is that CBF still increased with dopamine infusion in these awake sheep studies despite the presence of autoregulation (Myburgh et al., 2002), suggesting that dopamine does have a cerebrovascular dilatatory effect in addition to its inotropic action in increasing blood pressure. Nevertheless, cerebrovascular response in the immature brain may be different due to the ontogenic changes in cerebrovascular receptor sensitivity to catecholamines, which suggest that α-adrenergic receptor expression precedes that of β-adrenergic receptors and, finally, dopaminergic receptors (Von Essen, 1974; Edvinsson et al., 1979; Von Essen et al., 1980; Wagerle et al., 1990; Gleason et al., 2002) Wagerle et al. studied the pial arteriolar diameter decrease in response to norepinephrine and showed that preterm fetal lambs are significantly more sensitive to norepinephrine than full-term fetuses and newborn lambs, and that adult cerebral arterioles are refractory to the presence of norepinephrine (Wagerle et al., 1990). Gleason et al. also studied preterm fetal lambs and found that dopaminergic receptor blockade had no effect on the autoregulatory responses to dopamine, and the responses were completely blocked during α-adrenergic receptor blockade (Gleason et al., 2002). Hence, in the immature brain, it is possible that exogenous dopamine stimulates vasoconstrictor α-adrenergic receptors, and the vasodilator dopaminergic effects are minimal.

Nevertheless, studies using preterm and term newborn animal models, mainly lambs and piglets, have not shown that dopamine produces cerebral vasoconstriction or reduced CBF with dopamine infusion. Gleason et al. (2002) reported that in well and unanesthetized preterm and near-term fetal lambs, dopamine infusion up to 75 μg/kg/min caused no change in CBF and cerebral oxygen delivery despite an increase in MAP, suggestive of an appropriate cerebral autoregulatory response to the increased systemic blood pressure. Another study from the same group using a similar preterm fetal lamb model found that dopamine administration did not affect cerebral vasodilatory responses to severe hypoxia (Mayock et al., 2007).

A more recent study using newborn anesthetized piglets investigated the dose-dependant effects of five inotropes (dopamine, adrenaline, noradrenaline, dobutamine, and milrinone) on cerebral hemodynamic and oxygenation responses and identified significant differences in these responses to specific drugs and doses (Nachar et al., 2011). Low to medium doses of dopamine, adrenaline, dobutamine, and noradrenaline all increased blood pressure, common carotid blood flow, and cerebral oxygenation (measured by near infrared spectroscopy), whereas milrinone exerted minimal effects. However, this finding may in part be due to the anesthesia-induced impairment of cerebral autoregulation, leading to the parallel changes in blood pressure and CBF (Dagal and Lam, 2009). At higher doses, dopamine, adrenaline, and noradrenaline but not dobutamine decreased regional oxygenation in kidney, intestinal, and muscle, while cerebral oxygenation remained increased compared to baseline. The findings suggest an α-adrenoreceptor-mediated peripheral vasoconstriction resulting in a decrease in cardiac output and regional blood flow, but with a relative sparing of the cerebral vasculature from vasoconstriction. Consistent with a study in preterm infants (Pellicer et al., 2005), adrenaline also induced significant increases in muscle blood flow, serum glucose, and lactate concentrations in the newborn piglets, effects that would make it unfavorable as a first-line treatment. Another study comparing dopamine and dobutamine infusion in term and preterm piglets (Ferrara et al., 1995) showed that dopamine generated dose-dependent increases in CBF in both preterm and term piglet groups, an effect produced by dobutamine only in the term animals.

Overall, the results from animal studies are consistent with the findings of a recent meta-analysis showing increased CBF in preterm human studies receiving dopamine (Sassano-Higgins et al., 2011). No studies in preterm human infants or experimental preterm animal studies have demonstrated evidence that dopamine or adrenaline administration leads to excessive cerebro-vasoconstriction, reduced CBF or cerebral oxygenation.

### Cerebral metabolic rate with different inotropic agents

It is important to also consider cerebral metabolic rate when assessing the impact of changes in CBF. The balance between cerebral oxygen consumption and delivery determines the oxygen extraction and the overall cerebral tissue oxygenation. The effect of inotropic agents on cerebral metabolic rate in the preterm brain remains unknown. In the mature brain, it is generally accepted that monoamines (including catecholamines) do not cross the blood–brain barrier (BBB) (Emilien et al., 1999) and therefore have minimal influence on the cerebral tissue and cerebral metabolic rate (Myburgh et al., 2002). In studies using adult animal, when monoamines are administered in such a manner that the BBB is bypassed (by intraventricular injection or intracarotid infusion following induced BBB disruption), stimulation and increase of cerebral metabolism is noted and accompanied, probably secondarily, by a large increase in CBF (Mackenzie et al., 1976). Specific stimulation of cerebral dopaminergic receptors in adult baboons also results in increases in CBF which are always accompanied by increase in cerebral oxygen consumption (McCulloch and Harper, 1977), suggesting that the primary result of cerebral dopaminergic stimulation might be to increase cerebral metabolism, with CBF rising as a consequence of this metabolic effect.

Endogenous dopamine has also been proposed to play a fundamental role in the neurovascular regulation required for flow-metabolism coupling in the primate brain (Iadecola, 1998; Krimer et al., 1998). Association of dopamine therapy with an improved flow-metabolism coupling response has also been reported in a study in preterm infants (Wong et al., 2009). Accordingly, increase in cerebral perfusion with dopamine infusion may also be due to improved flow-metabolism coupling response in the preterm brain, in addition to the increased cerebral metabolic rate. These findings are relevant in the setting of intravenous dopamine therapy in preterm infants where there is evidence that the immature BBB allows access of exogenous dopamine to cerebral parenchyma (Seri et al., 1984). While the increase in CBF by dopamine reported in preterm infants may represent an impaired autoregulatory response to dopamine-induced increase in systemic blood pressure, the CBF rise may also be secondary to a dopamine-induced increase in cerebral metabolism as a flow-metabolism coupling response (Busija and Heistad, 1984). Similar mechanisms may also underlie the increase in CBF observed in preterm infants receiving adrenaline infusion (Pellicer et al., 2005) and would require further investigation.

While little is known about the effect of inotropic agents on cerebral metabolic rate in the preterm brain, dobutamine infusion was found to raise systemic metabolic rate and oxygen consumption in newborn lambs (1–2 days old, 7–8 days old, and 6–8 weeks old lambs) (Penny et al., 2001). The finding is consistent with studies in adults, in which catecholamines elevate the resting metabolic rate and systemic oxygen consumption (Chiolero et al., 1991). In newborn lambs receiving dobutamine infusion (Penny et al., 2001), systemic blood flow and oxygen delivery were elevated as well as increased oxygen consumption but was most apparent in 1–2 days old lambs. Importantly, this rise in oxygen consumption in 1–2 days old lambs was 7–12-fold greater than increases in the older lambs, outweighed the increase in systemic oxygen delivery and was associated with increase in oxygen extraction. It remains unknown whether similar effects of dobutamine and/or other catecholamines would also occur in the neonatal brain to raise cerebral metabolic rate and increase cerebral oxygen extraction; with such effects being most pronounced in the more immature brain of younger gestational age. This notion has important implications for the preterm brain injury, as offsetting the balance between cerebral oxygen consumption and delivery with high oxygen extraction may lead to cerebral hypoxia in the extreme case.

## Future research

There is a distinct lack of prospective research addressing cerebral effects of inotropic treatments in preterm infants and their long-term outcome (Shuhaiber et al., 2004). This is understandable given the ethical difficulty in setting up a clinical trial in which hypotensive preterm infants are randomized to receive either inotropic medications or placebo. While several animal and clinical studies have found short-term increase in CBF and cerebral oxygenation with use of inotropic medications, information of inotropic medications in relation to long-term outcome has mainly come from retrospective studies in infants treated for cardiovascular compromise. These are limited and as yet inconclusive due to the confounding effects of the more severe clinical illness and underlying pathologies in the hypotensive infants (Fanaroff et al., 2006); comparison with normotensive infants is clearly inappropriate. No randomized control study to explore effects of inotropic medications has been reported with untreated hypotensive infants as the control group, due to the obvious ethical concerns as mentioned above. Carefully designed research is needed to determine the long-term impact of different approaches to the treatment of neonatal hypotension and use of inotropic agents. Randomization of patients with hypotension but without clinical or laboratory evidence of cardiovascular compromise to have no inotropic treatment (permissive hypotension) (Dempsey et al., 2009) is a potential means to address the issue. Despite the application of preterm animal models to investigate preterm cerebral physiology and pathology, there have been no studies investigating the relationship between maintenance and/or increase of CBF with the administration of inotropes, to the neurological outcomes and histopathology in the developing brain. Such research is imperative given the widespread clinical use of inotropic medications in preterm infants, coupled with the scarce knowledge of its effects on the immature brain. Utilizing immature animal models to study and relate the neurophysiological and histopathological effects of inotropic therapy would fill this current knowledge gap and potentially influence clinical practice.

### Conflict of interest statement

The authors declare that the research was conducted in the absence of any commercial or financial relationships that could be construed as a potential conflict of interest.
